# Urinary Biomarkers of Exposure to Polycyclic Aromatic Hydrocarbons and Volatile Organic Compounds in San Joaquin Valley Families

**DOI:** 10.3390/toxics14100915

**Published:** 2026-10-16

**Authors:** Aalekhya Reddam, Marley Zalay, Rosemary Castorina, Asa Bradman, Daniel Sultana, Elizabeth Noth, Anabelle Garza, Debra Manzo Garcia, Tim Tyner, Jazmarie LaTour, Peyton Jacob, Josephine Alvaran DeGuzman, Jianwen She, Stephanie Jarmul

**Affiliations:** 1 Office of Environmental Health Hazard Assessment, California Environmental Protection Agency, Sacramento, CA 95812, USAstephanie.jarmul@oehha.ca.gov (S.J.); 2 Division of Environmental Health Sciences, School of Public Health, University of California, Berkeley, CA 94704, USArcastori@berkeley.edu (R.C.);; 3 Environmental Health Laboratory Branch, Center for Laboratory Sciences, California Department of Public Health, Richmond, CA 94804, USA; 4 Departments of Public Health and Environmental Systems and Health Sciences Research Institute, University of California, Merced, CA 95343, USA; abradman@ucmerced.edu; 5 Central California Asthma Collaborative, Fresno, CA 93727, USA; 6 Little Manila Rising, Stockton, CA 95206, USA; 7 Clinical Pharmacology Program, Division of Cardiology, Department of Medicine, University of California, San Francisco, CA 94143, USA

**Keywords:** polycyclic aromatic hydrocarbons (PAHs), volatile organic compounds (VOCs), urinary biomarkers, 2-hydroxynaphthalene

## Abstract

The San Joaquin Valley in California has some of the nation’s worst air quality. Health effects from elevated outdoor air pollution may be compounded by poor indoor air quality due to use of certain consumer products or individual activities. The BiomSPHERE study measured air pollution-related urinary exposure biomarkers in residents of Stockton and Fresno, California. Urine samples were collected from 64 adult–child pairs in 2023. Metabolites of four polycyclic aromatic hydrocarbons (PAHs) and five volatile organic compounds (VOCs) were measured in urine. Questionnaires were administered to collect information on demographics and potential exposure sources. Metabolites of all PAHs (fluorene, naphthalene, phenanthrene, and pyrene) and four VOCs (acrolein, acrylonitrile, crotonaldehyde, and propylene oxide) were detected in >85% of the samples. 2-Hydroxynaphthalene (2-NAP) was the only PAH metabolite that had higher concentrations in BiomSPHERE adults and children compared with nationally representative levels reported by the U.S. National Health and Nutrition Examination Survey (NHANES). Concentrations of 2-NAP were significantly correlated (*p* < 0.05) between adults and children, suggesting a common exposure source. Concentrations of 2-NAP were also significantly higher among Hispanic/Latino participants and among participants who recently used cleaning products, air fresheners, and scented personal care products (*p* < 0.05). For non-smoking adults, metabolites of the VOCs acrolein, acrylonitrile, and propylene oxide were significantly higher in BiomSPHERE compared with NHANES. Further research is needed to identify the sources of elevated 2-NAP and VOC urinary metabolite concentrations.

## 1. Introduction

The San Joaquin Valley (SJV) in California experiences some of the highest air pollution levels in the United States, often failing to meet federal standards. The California Assembly Bill 617 (AB 617) directed the California Air Resources Board to identify communities disproportionately impacted by air pollution and provide focused resources to mitigate exposures. Stockton and South Central Fresno in the SJV, California, were identified as AB 617 communities in 2019 and 2020, respectively [1]. Based on CalEnviroScreen, a geographically based program that ranks environmental exposures and socioeconomic stressors in all census tracts, the South Central Fresno community is among the worst 3% of census tracts in California, indicating a substantial cumulative impact burden [2].

Poor indoor air quality may compound the effects of outdoor air pollution in communities that already experience a disproportionate environmental and cumulative impact burden [3,4]. Individual activities such as consumer product use or cooking habits can contribute to reduced indoor air quality and increase exposure to harmful chemicals [5,6]. Therefore, it is important to examine how these products and activities can contribute to chemical exposures in these populations.

Two important classes of indoor and outdoor air pollutants are polycyclic aromatic hydrocarbons (PAHs) and volatile organic compounds (VOCs). PAHs are a class of semi-volatile compounds that can exist in both gas and particulate phases. The U.S. Environmental Protection Agency (EPA) has designated 16 PAHs as priority pollutants because of their toxicity and potential adverse effects on human health and the environment [7]. VOCs are gases emitted into the air from consumer products, industrial processes, and other sources. They can contribute to the formation of secondary air pollutants, including ground-level ozone and secondary organic aerosols, which are a component of fine particulate matter [8,9].

PAHs and VOCs can be generated from both outdoor and indoor sources. Outdoor sources of both pollutants include motor vehicle fuel combustion and industrial activities. Indoor sources include smoking or vaping, gas appliance use, high-temperature cooking methods, emissions from furnishings, and use of certain consumer products such as cleaning products, scented personal care products, and air fresheners [10,11,12]. Several studies have used biomonitoring to characterize personal exposure to PAHs and VOCs [8,13,14,15]; however, much of this research has focused on occupationally exposed populations and limited information on VOC exposure in general Californian communities is available. Furthermore, PAH and VOC exposures, measured as either ambient air levels or urinary metabolite levels, have been associated with adverse health outcomes such as increased cancer risk [16,17], neurological disorders [18], and all-cause mortality [19,20]. These findings underscore the need to better understand individuals’ exposures to these chemicals.

The goal of the biomonitoring component of the San Joaquin Valley Pollution and Health Environmental Research (BiomSPHERE) Study was to (1) evaluate PAH and VOC exposures in families living in Fresno and Stockton and (2) identify exposure patterns and potential indoor sources of these air pollutants among adults and children. To accomplish these goals, we measured biomarkers of four PAHs and five VOCs in urine samples from 64 families and tested hypotheses examining differences in biomarker levels between individuals, short-term temporal variability of the metabolite concentrations, comparisons with national reference levels, and associations between questionnaire responses and biomarker levels.

## 2. Materials and Methods

### 2.1. Participant Recruitment

An adult and child were recruited from 64 households in the Stockton and Fresno areas of California’s San Joaquin Valley. Eligible households included at least one child between 3 and 13 years of age and a parent or grandparent who spoke either Spanish or English. Families were recruited through community partners: the Central California Asthma Collaborative (CCAC) enrolled 52 households in Fresno, and Little Manila Rising (LMR) enrolled 12 households in Stockton. Participants were recruited through word of mouth and snowball sampling. In-home study visits were conducted by CCAC and LMR between February and November 2023. All study activities were approved by the Institutional Review Board at the University of California, Berkeley (Protocol ID 2021-06-14436). Adults provided written informed consent for themselves and for children under 7 years old, and children 7–13 years old provided assent to participate.

### 2.2. Data and Urine Sample Collection

Participant home visits took place over two consecutive days. On the first day, CCAC and LMR study staff administered a pre-sampling questionnaire to collect information about family characteristics, usual cooking practices, building characteristics (gas stove/heating, etc.), smoking, and other potential exposure determinants. Staff also provided participants with urine sample collection cups, small coolers, and icepacks. Study staff reviewed written instructions and provided links to a video explaining sample collection. One adult and one child in each of the participating families were asked to provide a first morning void urine sample the following morning and place samples in the cooler with frozen icepacks. Later that day, study staff retrieved the urine samples from participants’ homes and administered the post-sampling questionnaire, which collected specific information about potential exposure-related behaviors during the previous day and the morning before sample collection, such as household cleaning and consumer product use.

Participants were also asked if they were interested in participating in a sub-study which involved multiple days of urine sample collection. A subset of eight families provided daily first morning void samples over four consecutive days. Participants of the sub-study were provided with a refrigerator so that samples could be cooled immediately after collection.

All samples were retrieved by the study staff, transported on ice, and temporarily stored at CCAC and LMR in a −20 °C field freezer. Study staff prepared ten field blanks in the CCAC and LMR field offices by placing 40 mL of HPLC-grade water into the urine collection cups and storing them in the field freezer. Samples were periodically transported on dry ice to the Environmental Health Laboratory at the California Department of Public Health where they were processed, aliquoted, and analyzed for creatinine and specific gravity. Aliquots were stored at −80 °C until they were delivered on dry ice to the Clinical Pharmacology Laboratory at the University of California, San Francisco (UCSF) for analysis of PAH, VOC, and nicotine metabolites.

### 2.3. Measurement of Urinary Metabolites

In total, 174 urine samples (87 adult and 87 child samples) were analyzed for metabolites of PAHs, VOCs, and nicotine. Naphthalene metabolites included 1-hydroxynaphthalene (1-NAP) and 2-hydroxynaphthalene (2-NAP); fluorene metabolites included 1-hydroxyfluorene (1-FLUO), 2-hydroxyfluorene (2-FLUO), and 3-hydroxyfluorene (3-FLUO); phenanthrene metabolites included 1-hydroxyphenanthrene (1-PHEN), 2-hydroxyphenanthrene (2-PHEN), and 3- and 4-hydroxyphenanthrene (3&4-PHEN); and the pyrene metabolite was 1-hydroxypyrene (1-PYR). Metabolites of fluorene, phenanthrene, and pyrene were analyzed using liquid chromatography–tandem mass spectrometry (LC-MS/MS); detailed methods are described elsewhere [21]. In a separate analysis, urinary 1-NAP and 2-NAP were analyzed using gas chromatography–mass spectrometry (GC/MS) (a Thermo Trace 2000 GC with a 0.25 mm i.d. × 25 m DB-5 column (J & W Scientific, Folsom, CA, USA) interfaced to a Finnigan TSQ 7000 mass spectrometer (Thermo Fisher Scientific, San Jose, CA, USA)).

For VOCs, the acrylonitrile metabolite 2-cyanoethyl mercapturic acid (CNEMA), the propylene oxide metabolite 2-hydroxypropyl mercapturic acid (2-HPMA), the acrolein metabolite 3-hydroxypropyl mercapturic acid (3-HPMA), the crotonaldehyde metabolite 3-hydroxy-1-methyl-propyl mercapturic acid (HPMMA), and the benzene metabolite phenylmercapturic acid (PMA) were measured. These mercapturic acid metabolites were measured using HPLC-MS/MS using electron capture atmospheric pressure chemical ionization (ECAPCI) [22,23].

Metabolites of nicotine, free cotinine (COT) and free trans-3′-hydroxycotinine (3HC) were measured to account for exposures to cigarette smoke or e-cigarette vapors. These metabolites were measured using LC/MS as previously reported [24].

Quality control samples at four concentrations (70, 20, 1.5, and 0.75 ng/mL), each analyzed in triplicate, met acceptance criteria requiring at least 67% of all QCs and at least 50% of QCs at each level to fall within ±15% of nominal concentrations.

The limits of quantification (LOQ) for these metabolites were 0.025 ng/mL (nanogram per milliliter) for 1-FLUO, 2-FLUO, 3-FLUO, 1-PHEN, 2-PHEN, and 1-PYR; 0.05 ng/mL for 3&4-PHEN; 0.25 ng/mL for 1-NAP and 2-NAP; 1 ng/mL for 3-HPMA, HPMMA, and 2-HPMA; 0.5 ng/mL for CNEMA; 0.1 ng/mL for PMA; 0.05 ng/mL for COT and 0.1 ng/mL for 3HC (Table S1). All field blanks were below LOQ for all metabolites.

### 2.4. Statistical Analysis

Geometric means were calculated and statistical analyses performed for metabolites with detection frequencies above 65%. Values below the limits of quantification (LOQ) were imputed as LOQ/√2, consistent with the National Health and Nutrition Examination Survey (NHANES) methodology [25,26]. Metabolites of PAHs and VOCs were log-transformed to normalize the distributions. Two methods were used to adjust for urine dilution. Specific gravity adjustment was used for statistical analyses and associations with questionnaire data because it is considered a better measure for urine dilution, especially in children [27]. To compare BiomSPHERE participants with NHANES participants, we used creatinine-adjusted urinary metabolite concentrations from both datasets. Comparable data for each analyte from the most contemporaneous NHANES cycle were used to calculate summary statistics. To match the study age ranges, NHANES adults were restricted to 28–66 years old and NHANES children were restricted to 3–13 years old in the 2015–2016 and 2017–2018 cycles and 6–13 years old in the 2011–2012 cycles. NHANES did not recruit participants below 6 years old for the 2011–2012 cycle. NHANES weights, strata, and primary sampling units (PSUs) were incorporated when estimating the means, confidence intervals, and statistical comparisons using the *survey* package (version 4.6.1) in R.

Since source identification of urinary metabolites of PAHs and VOCs can be confounded by tobacco smoke exposure, we also compared levels in non-smoking NHANES participants with the levels in BiomSPHERE participants. Non-smoking was defined as participants having total serum cotinine ≤ 10 ng/mL for the 2011–2012 NHANES cycle and total urinary cotinine ≤ 91.7 ng/mL for the 2015–2016 and 2017–2018 NHANES cycles [28]. For BiomSPHERE participants, non-smoking was defined as participants having free urinary cotinine as <50 ng/mL [29]. Four adults in BiomSPHERE were classified as having elevated free urinary cotinine levels suggestive of tobacco smoking or nicotine vaping based on this threshold, out of which one adult reported smoking or vaping indoors. Weighted Student’s *t* test was used to identify significant differences between urinary metabolite levels in BiomSPHERE and NHANES.

We did not observe statistically significant differences in consumer product use (e.g., cleaning products) or urinary biomarkers biomarker concentrations between Fresno and Stockton participants. The proportion of participants who identified as Hispanic/Latino differed between the two communities, and race/ethnicity was included as a covariate in the regression models. Given the substantially smaller Stockton sample, these comparisons had limited power to detect geographic differences. Participants from both communities were therefore combined for the primary analyses. We calculated Pearson correlations between analyte concentrations within urine samples and between paired adult and child urinary metabolite concentrations. For families who provided multiple urine samples, for most analyses of urinary metabolite data, we used metabolite concentrations from the sample collected closest in time to the post-sampling questionnaire, typically on the same morning. Since the half-lives of chemicals are on the order of 7–12 h, it was most appropriate to use the sample collected the closest in time to the post-sampling questionnaire to capture the most relevant exposure. Repeated urine samples were provided by eight families; seven families provided urine samples on four consecutive days, and one family provided urine samples on three days. We evaluated the short-term variability of the urinary biomarkers within these participants using intraclass correlations coefficients (ICCs), and determined the total, within-, and between-participant variability using a random effects model. An ICC of ≥0.75 indicates excellent repeatability, a value between 0.4 and 0.75 indicates fair repeatability and an ICC < 0.4 indicates poor repeatability [30].

We used multivariable linear regression models to evaluate associations between participant product use, housing characteristics, and activities reported in questionnaires and metabolites of PAHs and VOCs separately for adults and children. Additional details on the variables measured are reported in the Appendix A. We summed all fluorene metabolites (∑FLUO = 1-FLUO + 2-FLUO + 3-FLUO) and phenanthrene metabolites (∑PHEN = 1-PHEN + 2-PHEN + 3&4-PHEN). Models were adjusted for race/ethnicity as a social construct representing institutionalized differences that can systemically shape environmental exposures [31,32]. Because the majority of BiomSPHERE adult participants identified as Hispanic/Latino, and sample sizes for other racial/ethnic groups were much smaller, race/ethnicity was categorized as Hispanic/Latino or non-Hispanic/Latino for the analysis. Participants who identified with multiple racial/ethnic groups were classified as Hispanic/Latino if any reported race/ethnicity was Hispanic/Latino. Children’s race/ethnicity was assigned based on the race/ethnicity of the corresponding adult participant. Models were also adjusted for free cotinine, a metabolite of nicotine, which may indicate tobacco smoke exposure, an important source of some PAHs and VOCs. Cotinine levels were adjusted for specific gravity and log-transformed for normality. In addition, we also adjusted for covariates that were predictors of at least one urinary metabolite: age (in years) and household income (categorical, Table 1). We selected covariates associated with at least one metabolite to keep a common covariate set across the models. To account for multiple comparisons in the exploratory analyses of questionnaire variables and urinary metabolites, we performed sensitivity analyses using the Benjamini–Hochberg procedure to control the false discovery rate (FDR) at q < 0.05.

Because we identified significantly higher urinary 2-NAP concentrations in Hispanic/Latino participants in BiomSPHERE (see Section 3), we conducted additional analyses to evaluate these differences. Ethnicity categories differed slightly between studies: NHANES classified participants as Mexican American/Other Hispanic whereas BiomSPHERE classified participants as Hispanic/Latino. We used weighted *t* tests to compare 2-NAP concentrations between Hispanic/Latino and White participants in BiomSPHERE, between Mexican American/Other Hispanic and White participants in NHANES, and between BiomSPHERE and NHANES within each racial/ethnic group. Race/ethnicity subgroups other than Hispanic/Latino in BiomSPHERE were too small to assess effect modification. We examined percent of product use in Hispanic/Latino and Not-Hispanic/Latino participants and associations between product use and urinary 2-NAP concentrations within the Hispanic/Latino subgroup. All statistical analyses were completed using R version 4.3 (Vienna, Austria) and statistical significance was assessed at alpha = 0.05.

## 3. Results

### 3.1. Descriptive Statistics

Adults had a mean age of 42 years (range: 28–66), and children had a mean age of 9 years (range: 3–13) (Table 1). Most adults were female (97%), compared with 45% of children. Participants were predominantly Hispanic/Latino (69%), and nearly half (48%) reported Spanish as their primary language. Most participants lived in Fresno (81%), and more than half (56%) reported a household income of less than $30,000.

Table 2 presents descriptive statistics of the specific gravity-adjusted concentrations of urinary metabolites in adults and children. Unadjusted and creatinine-adjusted concentrations are presented in Table S1 and Table S2, respectively. In both children and adults, PAH and VOC metabolite detections were widespread (Table 2; Tables S1 and S2). The most commonly detected PAH metabolites were 2-NAP (DF = 100%), as well as 2-FLUO, 1-PHEN, and 1-PYR (DF > 85%). Of the PAH metabolites, 2-NAP had the highest geometric mean concentrations in both adults and children (16,000 ng/L and 13,000 ng/L, respectively). Among VOC metabolites, 3-HPMA, HPMMA, 2-HPMA, and CNEMA were detected in more than 90% of samples, and PMA was detected in 31% of samples. The acrolein metabolite, 3-HPMA, had the highest geometric mean concentrations in both adults and children (270 ng/mL and 320 ng/mL, respectively) followed by HPMMA, a metabolite of crotonaldehyde (110 ng/mL in adults and 140 ng/mL in children). COT was detected in 66% and 72% of adults and children respectively and 3HC was detected in 88% of adults and children; both are metabolites of nicotine.

### 3.2. Comparison with NHANES

**Figure 1:**
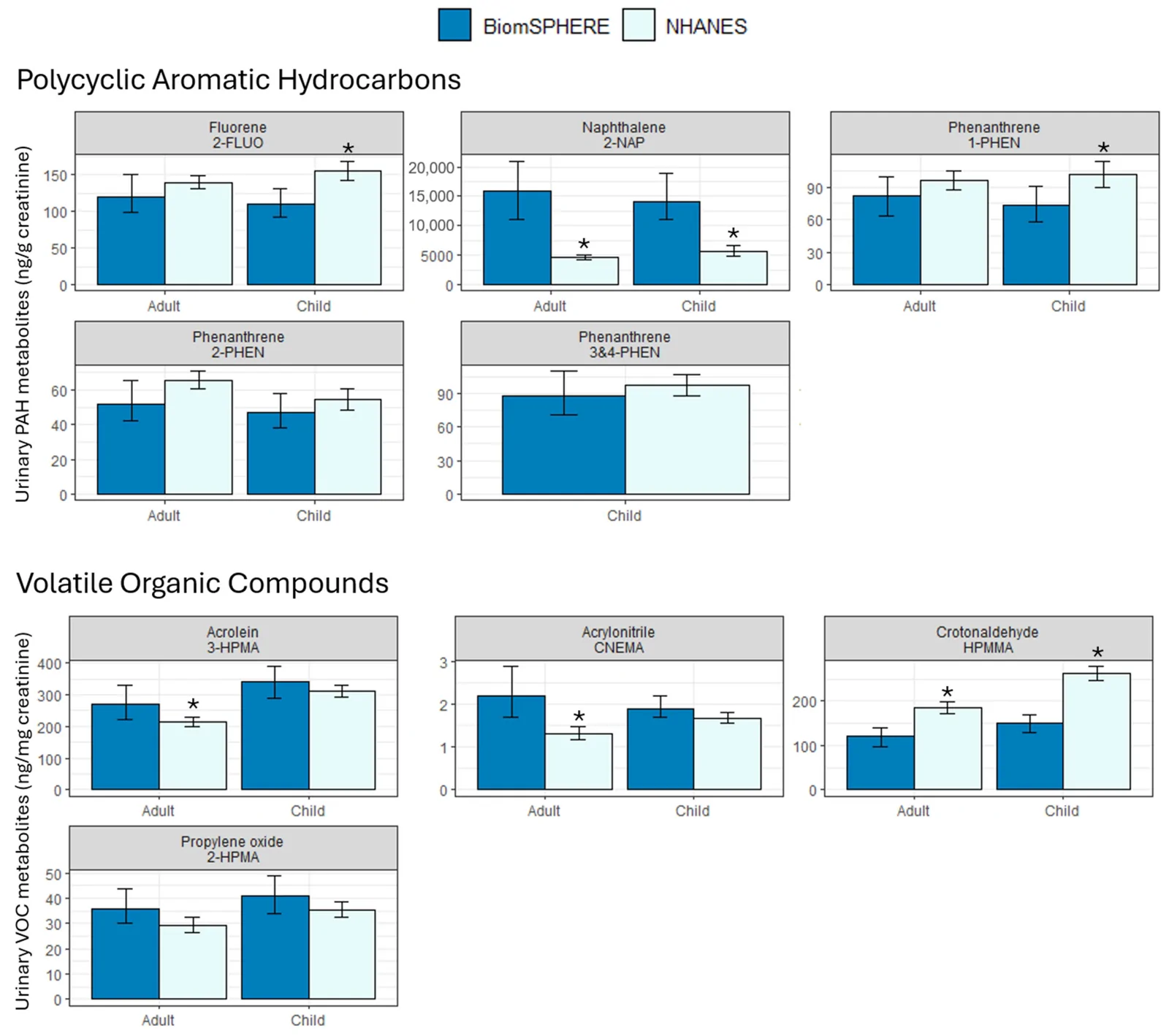
Geometric means (GM) and 95% confidence intervals (CIs) of PAH and VOC metabolite concentrations in non-smoking participants (N = 60 adults; N = 64 children) in BiomSPHERE and NHANES. * represents a significant difference (*p*-value < 0.05) between BiomSPHERE and NHANES. Source for 2-FLUO, 1-PHEN, 3-HPMA, CNEMA, HPMMA, and 2-HPMA: NHANES 2017–2018; source for 2-NAP: NHANES 2015–2016; source for 2-PHEN and 3&4-PHEN: NHANES 2011–2012. GM and 95% CIs were not calculated for 3&4-PHEN in adults and 1-PYR in adults and children because detection frequency was below 65% in BiomSPHERE and NHANES respectively. Non-smoking was defined as participants having total serum cotinine ≤ 10 ng/mL for the 2011–2012 NHANES cycle and total urinary cotinine ≤ 91.7 ng/mL for the 2015–2016 and 2017–2018 NHANES cycles. For BiomSPHERE participants, non-smoking was defined as participants having free urinary cotinine as <50 ng/mL.

Among PAHs, 2-NAP levels were statistically significantly higher in BiomSPHERE participants compared with NHANES participants in both adults and children. The geometric means of the other PAH metabolites were either similar to or lower than geometric means in NHANES (Table S3 and Figure S1). Results are similar when the BiomSPHERE and NHANES study populations are restricted to non-smokers (Figure 1).

Among VOCs, levels between the BiomSPHERE and NHANES participants were similar (Figure S1). However, when restricted to non-smoking adults, 2-HPMA, 3-HPMA, and CNEMA were significantly higher in BiomSPHERE than in NHANES (Figure 1).

Levels of 2-NAP in BiomSPHERE were significantly higher in Hispanic/Latino participants compared with White participants (Figure S2). In NHANES, levels of 2-NAP were also significantly higher in Mexican American or Other Hispanic adults compared with White adults. However, levels of 2-NAP were significantly higher in the Hispanic/Latino participants in BiomSPHERE than the Mexican American or Other Hispanic participants in NHANES. In contrast, the levels of 2-NAP were similar in White adults in BiomSPHERE compared with White adults in NHANES.

### 3.3. Correlations of PAHs and VOCs in BiomSPHERE

**Figure 2:**
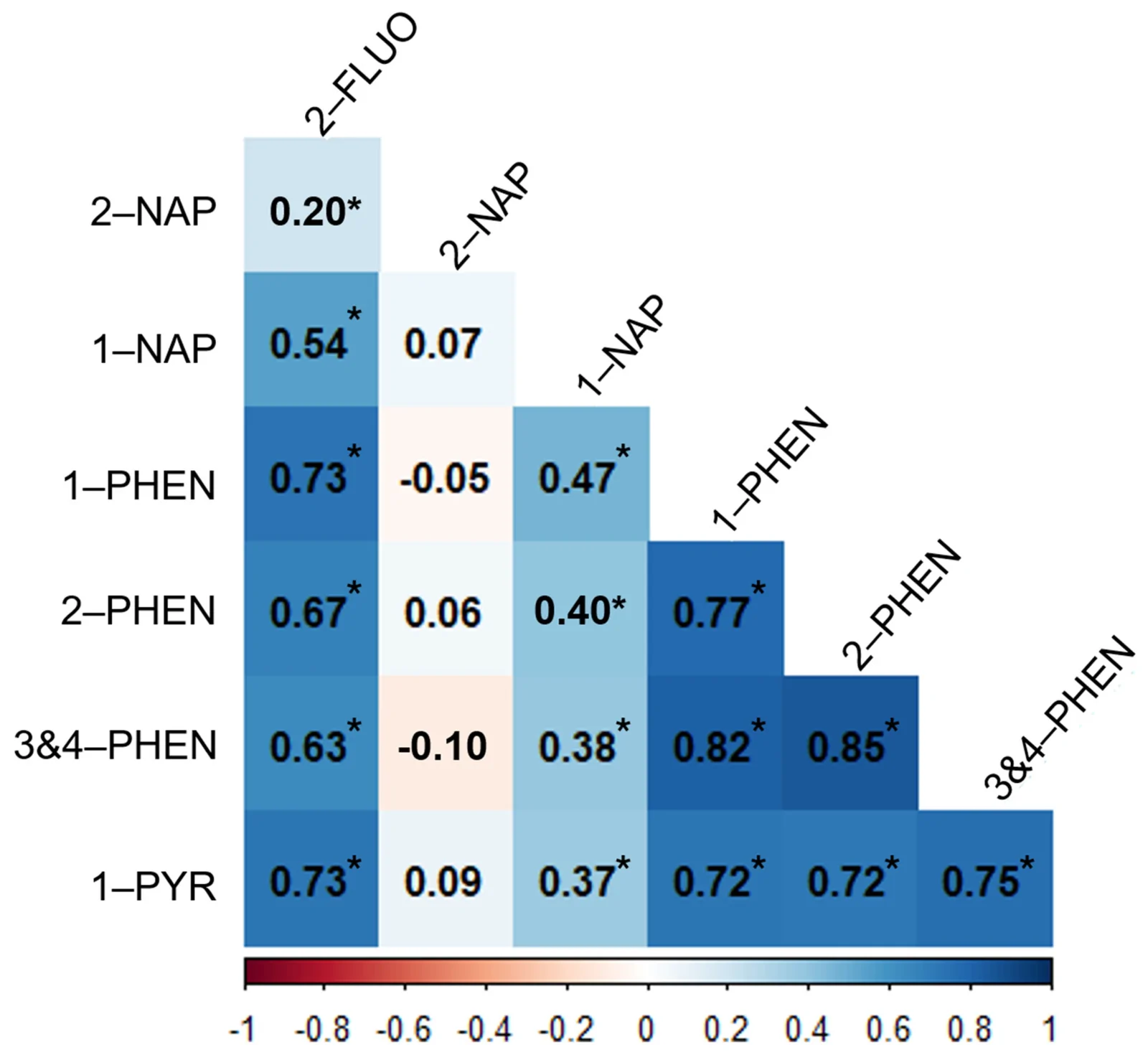
Pearson correlation coefficients of specific gravity adjusted and log-transformed PAHs in BiomSPHERE (adults and children). * represents a statistically significant association (*p* < 0.05).

Most PAH metabolites were strongly correlated with each other (r = 0.63–0.85) with the exception of 1-NAP and 2-NAP (Figure 2). 1-NAP was only moderately correlated with the other PAH metabolites (r = 0.37–0.54). 2-NAP was weakly correlated only with 2-FLUO and was not correlated with any of the other PAH metabolites suggesting a unique exposure source of 2-NAP. All VOC metabolites were moderately correlated with each other (r = 0.36–0.56), with the exception of CNEMA with HPMMA (Figure S3).

**Figure 3:**
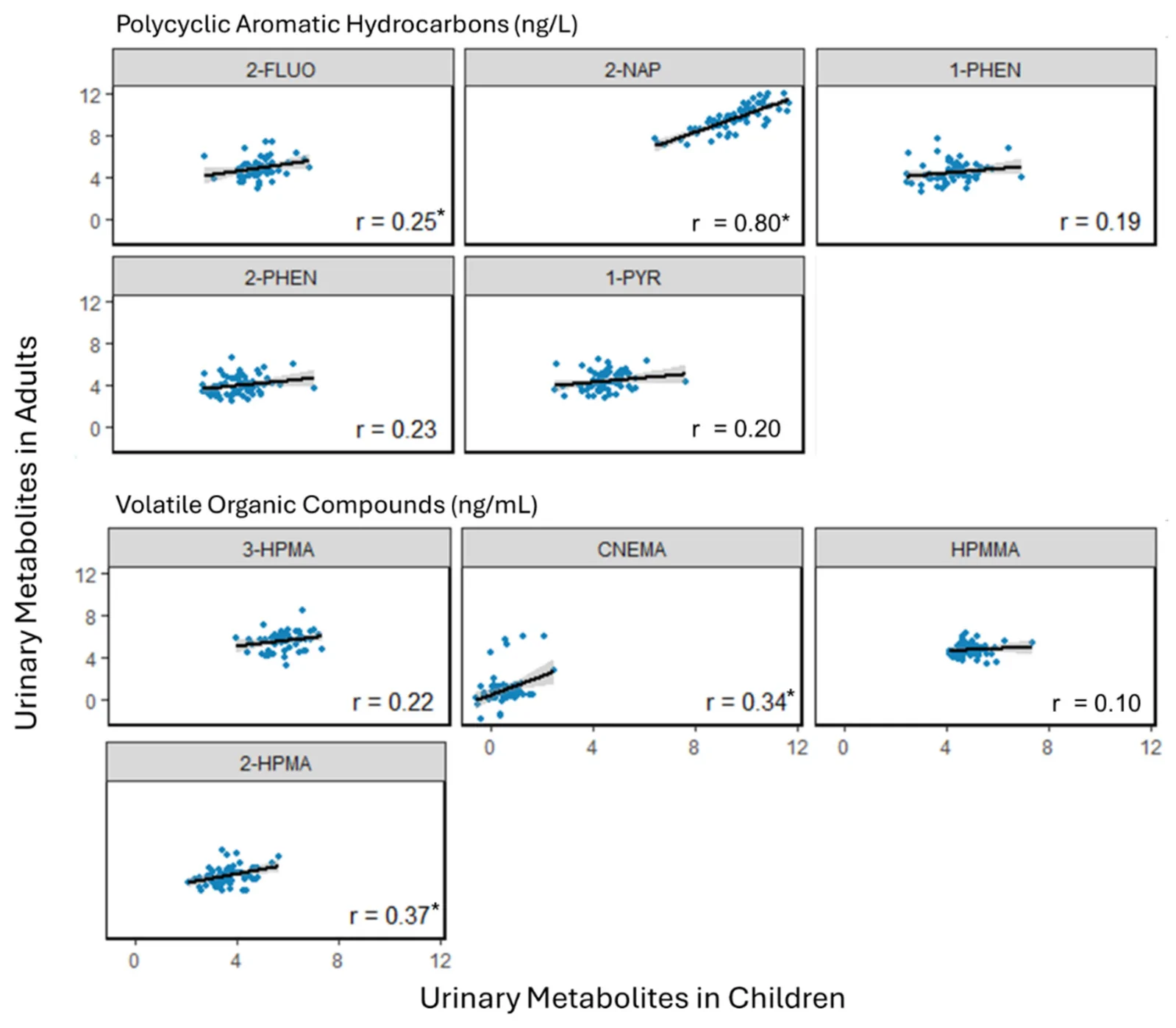
Correlations of PAH and VOC metabolites between BiomSPHERE adults (*y*-axis) and children (*x*-axis). Metabolites are log-transformed and specific gravity-adjusted. r is the Pearson correlation coefficient. * represents a statistically significant association (*p* < 0.05).

Among all urinary metabolites, 2-NAP had the strongest correlation between adults and children (r = 0.80). 2-FLUO (r = 0.25), 2-HPMA (r = 0.37), and CNEMA (r = 0.34) were also significantly, albeit weakly, correlated between adults and children in BiomSPHERE (Figure 3).

We also examined the within- and between-participant variability of the urinary metabolites from samples collected on up to four consecutive days in adults (N = 31) and children (N = 31) from 8 families. When examining the variance between and within participants, we observe a lower within-participant variance compared to between-participant variance for 2-NAP (adult ICC = 0.94, child ICC = 0.88), 3-HPMA (adult ICC = 0.72, child ICC = 0.53), and CNEMA (adult ICC = 0.89, child ICC = 0.50) in adults and children and 2-FLUO (ICC = 0.84), 1-PYR (ICC = 0.79), HPMMA (ICC = 0.75), and 2-HPMA (ICC = 0.60) in adults (Table S4). This suggests that an individual’s exposures to the PAHs and VOCs excreted as these urinary biomarkers are relatively stable. This finding contrasts with many studies showing high within-subject variability for urinary metabolite exposure biomarkers [33,34]. Based on the ICCs, 2-NAP was the only metabolite with excellent repeatability in adults and good repeatability in children (Table S4).

### 3.4. Associations of Urinary Metabolite Concentrations with Household Information

#### 3.4.1. PAHs

Use of various consumer products was significantly and positively associated with urinary levels of 2-NAP (Table 3). The uses of any air fresheners (adults β = 1.80, children β = 2.16) and plug-in air fresheners (adults β = 3.69, children β = 3.08) were associated with higher levels of 2-NAP in adults and children, with use of air freshener spray also significant in children (β = 1.95). The number of cleaning products used (adults β = 1.20, children β = 1.21) and the use of all-purpose spray or aerosol cleaners (adults β = 2.79, children β = 2.34) and bleach-containing products (adults β = 1.77, children β = 1.81) were also significantly associated with 2-NAP levels in adults and children. Significant positive associations were seen between the use of perfume (β = 2.20) and scented body lotion (β = 1.98) with 2-NAP levels in adults. We also observed non-significant positive associations between air freshener sprays and 2-NAP in adults and cleaning products (floor cleaner, glass cleaner, and restroom cleaners) and 2-NAP in both adults and children (Table 3). Significant inverse associations were observed between other PAH metabolites and use of air freshener products and certain cleaning products (bleach-containing products, all-purpose spray, floor cleaner, and disinfectant wipes) (Table S5).

We observed significant positive associations for gas stove use and significant negative associations for electric stove use with ∑FLUO and ∑PHEN metabolites in children. We also observed similar non-significant associations with gas and elective stove use and ∑FLUO, ∑PHEN, and 1-PYR metabolites in adults (Table S5). Only the associations between plug-in air freshener use and 2-NAP levels remained significant in adults and children after using the Benjamini–Hochberg false discovery rate procedure.

#### 3.4.2. VOCs

We observed significant positive associations between glass cleaner use with CNEMA in adults (β = 2.25). We also found significant inverse associations between the number of cleaning products used (β = 0.89) and floor cleaner (β = 0.57) with 3-HPMA and use of any air freshener with CNEMA in adults (β = 0.57). Lastly, significant negative associations between glass cleaner (β = 0.60) and disinfectant wipe use with 2-HPMA (β = 0.65) in children were observed (Table S6). No associations remained significant in adults and children after using the Benjamini–Hochberg false discovery rate procedure.

#### 3.4.3. Associations of Urinary 2-NAP Concentrations with Race/Ethnicity-Specific

Hispanic/Latino participants were nearly twice as likely to report any air freshener, perfume, and scented lotion use (Table S7). The use of plug-in air fresheners, the number of cleaning products, and bleach-containing products were associated with 2-NAP levels in Hispanic/Latino children and adults. Use of all-purpose cleaners and perfumes was also significantly associated with 2-NAP levels in Hispanic/Latino adults.

## 4. Discussion

### 4.1. Levels of PAH and VOC Urinary Metabolites

Overall, urinary 2-NAP levels were significantly higher in BiomSPHERE participants compared with the NHANES reference population. Among non-smoking adults, urinary metabolites of acrolein, acrylonitrile, and propylene oxide were also significantly higher in BiomSPHERE participants than in NHANES.

This study adds to the limited research examining PAH and VOC exposure in residents of California’s San Joaquin Valley. The Center for the Health Assessment of Mothers and Children of Salinas (CHAMACOS) cohort collected urine samples from pregnant individuals in the Salinas Valley from 1999 to 2000. This population was similar to BiomSPHERE participants as they were predominantly Hispanic/Latino women in lower income communities living in California agricultural regions. The 2-NAP levels reported in CHAMACOS were approximately 10 times lower than concentrations in BiomSPHERE (unadjusted 2-NAP concentration of 1700 ng/L) [35]. In contrast, CHAMACOS reported an unadjusted 75th percentile of 1-NAP of 2900 ng/L, which was higher than the corresponding percentile observed in BiomSPHERE. The Stockton Air Pollution Exposure Project (SAPEP) collected urine samples from children in 2021 and reported a median concentration of 19,000 ng/g creatinine for combined 1- and 2-NAP that was similar to the BiomSPHERE participants [23]. Compared to populations studied in other regions of California between 2005–2019 [36,37,38,39], urinary concentrations of 2-NAP were significantly higher in BiomSPHERE. Other PAH metabolite concentrations in these studies were generally similar to those observed in BiomSPHERE [23]. Among other studies in the U.S., we were only able to identify one that reported urinary 2-NAP concentrations similar to levels in BiomSPHERE: female hairdressers in Maryland and Washington D.C. between 2018–2019 had a geometric mean concentration of 17,400 ng/L, which was higher than BiomSPHERE [40].

VOC metabolite concentrations in SAPEP were also generally similar to those observed in BiomSPHERE. Other than SAPEP, we were unable to find any other published values of VOC metabolites in Californian residents. Outside of California, pregnant individuals across seven cities in the U.S. who reported comparable indoor air exposures had similar or slightly higher VOC concentrations compared to BiomSPHERE participants [15].

The lower levels of PAH metabolites other than 2-NAP in BiomSPHERE participants compared to NHANES may partially reflect lower levels of PAHs as air pollutants in the SJV during the sampling season (spring–fall). Research has shown a decrease in particle bound PAHs in the San Joaquin Valley from 2000–2019, likely attributed to a decline in agricultural and biomass waste burning [41]. A study by Knox et al. found that urinary levels of 2-FLUO, 1-PHEN, 3-PHEN, 2-NAP, and 1-PYR were lower in Californians compared to the rest of the U.S. population between 2001 and 2014 [42]. Interestingly, while levels of 2-FLUO, 1-PHEN, and 3-PHEN significantly decreased over time, urinary concentrations of 2-NAP significantly increased for both Californians and the United States between 2001–2014 [42].

Similar to our study, higher urinary levels of CNEMA, a metabolite of acrylonitrile, and 2-HPMA, a metabolite of propylene oxide, were observed in Californians compared with the U.S. in the study by Knox et al. [42]. We additionally saw significantly higher levels of 3-HPMA, a metabolite of acrolein, in BiomSPHERE compared to levels in NHANES. Limited research on potential sources of acrolein, acrylonitrile, and propylene oxide in the SJV limits our ability to explain the differences we observed. Furthermore, more recent national data is needed to determine if the elevated levels of some PAH and VOC metabolites in BiomSPHERE could be a reflection of temporal trends across the country.

### 4.2. Correlations of PAHs and VOCs

The correlation of 2-NAP with other PAHs has been mixed among other studies and in NHANES. A study by Dobraca et al. and NHANES report moderate correlations of 2-NAP with other PAHs [36,43]. Other studies analyzing samples collected more recently in California report trends similar to BiomSPHERE, suggesting that 2-NAP has a unique exposure source in these populations [36,39,44]. Interestingly, 2-NAP is not correlated with 1-NAP in BiomSPHERE, suggesting that exposure might be directly from 2-NAP used in a product, or indirectly from a chemical other than naphthalene that is metabolized to 2-NAP. However, the relatively low detection frequency limits interpretation of associations involving 1-NAP, including its correlation with 2-NAP.

In our population, 2-NAP had the strongest correlation between adults and children, suggesting a common exposure source that is not specific or unique to work or school. Additionally, 2-NAP was the only urinary analyte that had excellent repeatability in adults and good repeatability in children. Since the half-life of 2-NAP is short (~12 h), this finding suggests a relatively consistent source of exposure. However, due to the sample size of eight adult–child pairs and a daily sampling duration of four days, these results should be interpreted with caution.

### 4.3. Associations Between PAH Metabolite Concentrations with Household Information

We observed significant positive associations for gas stove use and significant inverse associations for electric stove use with ∑FLUO and ∑PHEN metabolites in children and similar non-significant associations in adults. Studies have shown that cooking on a gas stove compared to an electric stove releases higher levels of PAHs, aldehydes, and particulate matter suggesting that stove electrification may improve indoor air quality [45,46,47].

We also observed significant inverse associations between use of cleaning products and ∑FLUO, ∑PHEN, and 1-PYR. A small percentage of fluorene and phenanthrene and a larger percentage of pyrene can be particle-bound and therefore one hypothesis could be that the use of cleaning products may remove particle-deposited PAHs in dust and prevent resuspension into air. However, more research is needed to confirm these findings in a residential setting with environmental measures.

We observed significant positive associations with 2-NAP and cleaning products, air fresheners, and personal care product use. Unlike the other PAHs that may partition between gas phase and particle phase, naphthalene primarily exists in the gas phase, which might partially explain the difference in results. Naphthalene has been found in products such as toilet deodorant products, moth balls, and air fresheners [48,49,50]. Building materials and furnishings have also been found to be a significant source of indoor naphthalene, particularly polyvinyl chloride (vinyl) and wood furniture and painted walls and ceilings [51]. Naphthalene is used in a variety of industrial processes to make consumer products containing phthalate plasticizers, synthetic resins, paint, and polyvinyl chloride [52]; thus, is it possible that products in the home contribute to 2-NAP concentrations. We also observed a larger effect size for the use of plug-in air fresheners compared to air freshener sprays in relation to urinary 2-NAP levels. The difference may be related to different use patterns: air freshener sprays are generally used more intermittently, whereas plug-in air fresheners are typically used continuously, resulting in prolonged exposure.

Concentrations of 2-NAP were positively associated with several fragranced products in our study. However, since products containing fragrances are not required to list the individual components of those scents on labels in the U.S. [53], we were unable to determine which fragrances could contain 2-NAP, naphthalene, or other chemicals that may be metabolized to 2-NAP. To date, there is no current evidence showing associations between fragranced products and elevated levels of 2-NAP. In a study comparing chemical exposures in pregnant individuals, participants in Fresno had a higher number of chemicals annotated as food additives, cosmetics, colorants, and fragrances compared with participants in San Francisco [54]. Additionally, higher frequency of perfume, cologne, or scented body spray use has been observed in Hispanic/Latinx and Black women compared to White women in California [55]. We also observed a similar trend, where Hispanic/Latino participants in BiomSPHERE were nearly twice as likely to use perfume and scented lotion compared to Not-Hispanic/Latino participants.

We observed significantly higher levels of 2-NAP in BiomSPHERE Hispanic/Latino participants compared to White participants which is consistent with what is found in NHANES as well as other studies [17,36]. This pattern may reflect socioeconomic or structural factors such as housing conditions, neighborhood characteristics, and access to or use of consumer products that are unequally distributed across racial and ethnic groups rather than ethnicity itself as an exposure determinant. We did not find evidence that associations of cleaning products, air fresheners, and personal care products with 2-NAP differ in Hispanic/Latino participants compared to Non-Hispanic/Latino participants due to the small sample number of participants in the other racial/ethnic groups. These findings need to be further investigated with larger sample sizes.

While other studies have found significant associations between concentrations of urinary PAH metabolites and grilled or smoked food consumption [37,56,57] and the presence of an attached garage [58], we did not identify any significant associations between these variables and PAH metabolites in BiomSPHERE participants.

### 4.4. Associations of VOC Metabolite Concentrations with Household Information

We only observed a significant positive association between glass cleaner use with CNEMA in adults. We did not see significant positive associations between other consumer products, consumption of grilled food, type of stove used (gas vs. electric), and presence of attached garage with VOC metabolites. Past research has identified positive associations between residential sources such as paint and solvents, gas stoves, and gasoline components in attached garages with benzene, toluene, ethylbenzene, and xylene [15,59,60]. Metabolites of most of these VOCs were chemicals not measured in the BiomSPHERE population and the one metabolite of benzene that was measured, PMA, had a very low detection frequency.

### 4.5. Limitations and Future Directions

Limitations of this study include the small sample size, which may have reduced statistical power to identify other exposure sources or differences by demographic groups due to the small number of participants within each subgroup. This study was conducted in a specific region of California, and the recruitment strategies may have the potential for selection bias; therefore, the results may not be representative of the Fresno, Stockton or San Joaquin Valley communities or generalizable to the general population. Therefore, the findings may reflect socially patterned exposures and require confirmation in larger and more diverse populations.

Although most metabolite concentrations were comparable with national levels, comparisons with NHANES and other studies may have been confounded by temporal trends, differences in the timing of sample collection, sampling protocols, and demographic composition. Moreover, given the cross-sectional nature of the study, it is not known whether these concentrations are representative of what would be collected at a different point in time.

Significant associations between urinary biomarkers and questionnaire data could be by chance due to the small sample size and multiple comparisons, and further research in larger sample sizes is needed to validate these exploratory analyses. Additionally, while we collected information about a wide range of consumer product use and daily activities, this study was not specifically designed to characterize exposure to 2-NAP, and we may not have captured other important exposure sources, such as diet or other consumer products (i.e., moth balls). We also didn’t ask about the number of times a product was used so were unable to identify the impact of the magnitude of exposure. Moreover, we were unable to identify specific ingredients in fragranced consumer products. Future studies are needed to examine chemical compositions of these products and identify chemicals that might contribute to elevated 2-NAP levels. Urinary biomarkers reflect internal dose and typically cannot distinguish between outdoor and indoor air, and dietary sources of exposure.

Asphalt or bitumen, a common component of road paving, emits 2–3-ring PAHs such as naphthalene [61]. While we did not identify any clusters of asphalt production sites near our participants, further research is needed to examine the contribution of recent road paving and other asphalt sources to urinary 2-NAP levels.

Smoking status was defined using different biomatrices and cotinine measures for different NHANES cycles and in BiomSPHERE which might affect comparability.

1-NAP was not included in descriptive analyses because only 51% of urine samples had detectable concentrations, possibly due to an analytical interference based on the sulfatase activity from the beta-glucuronidase enzyme used to release the free hydroxylated metabolites [62], which limits interpretation of its correlation with 2-NAP. Lastly, although we adjusted for relevant confounders in our analyses, the observed associations may still have been affected by unmeasured factors, such as lifestyle characteristics or air pollutant exposures occurring outside the home.

BiomSPHERE also included an air monitoring component where air pollutants were measured indoors and outdoors at the participants’ home over a 24-h sampling period. Future research will examine contributions of indoor and outdoor air pollution concentrations to urinary metabolite levels which would further clarify the source of urinary metabolites.

## 5. Conclusions

This study measured biomarkers of PAH and VOC exposure in adults and children in the San Joaquin Valley. We found significantly higher concentrations of metabolites of acrolein (3-HPMA), acrylonitrile (CNEMA), and propylene oxide (2-HPMA) in non-smoking adults in BiomSPHERE compared with NHANES. With the exception of 2-NAP, we found that most urinary PAH concentrations were similar to or lower than those reported in NHANES and other studies. Concentrations of 2-NAP were approximately 3 times higher in BiomSPHERE compared with NHANES with significantly higher concentrations in Hispanic/Latino participants compared to White participants. Correlations between adults and children and intraclass correlations suggest that there is a common and relatively consistent source of 2-NAP exposure. Furthermore, non-significant correlations between 2-NAP and 1-NAP suggest that 2-NAP levels might be from exposure to 2-NAP itself or another chemical that metabolizes to 2-NAP, rather than from naphthalene alone. We also identified significant positive associations between use of household cleaning products, air fresheners, and perfumes and 2-NAP concentrations in both adults and children. Given that 2-NAP, 3-HPMA, CNEMA, and 2-HPMA have been associated with a range of adverse health outcomes and biological effects, this study highlights the need for further research to better understand the sources of personal exposure contributing to elevated urinary levels of these chemicals.

## Figures and Tables

**Table 1:** Demographic characteristics of adults (N = 64) and children (N = 64) in BiomSPHERE (San Joaquin Valley, CA, USA, 2023).

Characteristic	Characteristic Breakdown	N (%)/ Mean ± SD
Age of Child Participants (years)	-	8.8 ± 2.6
Age of Adult Participants (years)	-	41.9 ± 7.6
Adult Sex Assigned at Birth	Male	2 (3%)
	Female	62 (97%)
Child Sex Assigned at Birth	Male	33 (52%)
	Female	31 (48%)
Race/Ethnicity of Adult	Hispanic/Latino	44 (69%)
	White	8 (12%)
	Black/African American	3 (5%)
	Asian	2 (3%)
	Multiple Races/Ethnicities	7 (11%)
Language Spoken by Adult	English	33 (52%)
	Spanish	31 (48%)
Household Annual Income	Less than $15,000	11 (17%)
	$15,000 to $30,000	25 (39%)
	$30,000 to $50,000	9 (14%)
	$50,000 to $75,000	4 (6%)
	More than $75,000	15 (23%)
Rent or Own Home	Rent	35 (55%)
	Own	29 (45%)
City of Residence	Fresno	52 (81%)
	Stockton	12 (19%)

**Table 2:** Descriptive statistics of specific gravity-adjusted urinary metabolite concentrations (ng/L) of PAHs and VOCs in adults (N = 64) and children (N = 64) in BiomSPHERE.

Parent Compound	Metabolite	Adult/ Child	DF (%)	Geometric Mean (95% CI)	Median (IQR)	Maximum
Polycyclic Aromatic Hydrocarbons (ng/L)						
Fluorene	1-FLUO	Adult	52	NC	28 (<LOQ-59)	990
		Child	47	NC	<LOQ (<LOQ-44)	690
	2-FLUO	Adult	94	130 (100–160)	110 (70–180)	1700
		Child	92	100 (87–120)	99 (71–150)	720
	3-FLUO	Adult	45	NC	<LOQ (<LOQ-44)	1300
		Child	48	NC	<LOQ (<LOQ-39)	720
Naphthalene	1-NAP	Adult	53	NC	290 (<LOQ-570)	13,000
		Child	44	NC	<LOQ (<LOQ-500)	5500
	2-NAP	Adult	100	16,000 (11,000–22,000)	15,000 (7200–38,000)	160,000
		Child	100	13,000 (10,000–18,000)	16,000 (6900–34,000)	110,000
Phenanthrene	1-PHEN	Adult	91	83 (66–110)	76 (46–130)	2200
		Child	86	68 (55–85)	70 (40–110)	1000
	2-PHEN	Adult	69	52 (41–65)	44 (<LOQ-97)	750
		Child	69	44 (35–54)	42 (<LOQ-78)	890
	3-&4- PHEN	Adult	56	NC	72 (<LOQ-120)	1300
		Child	72	82 (67–100)	81 (<LOQ-120)	1700
Pyrene	1-PYR	Adult	88	78 (63–98)	69 (40–150)	650
		Child	88	80 (65–98)	76 (53–130)	1900
Volatile Organic Compounds (ng/mL)						
Acrolein	3-HPMA	Adult	100	270 (220–340)	300 (190–430)	5000
		Child	100	320 (270–380)	320 (210–530)	1400
Acrylonitrile	CNEMA	Adult	92	2.6 (1.8–3.9)	1.8 (1.4–3.4)	420
		Child	94	1.8 (1.6–2.1)	1.8 (1.2–2.6)	12
Benzene	PMA	Adult	31	NC	<LOQ (<LOQ-0.16)	4.1
		Child	31	NC	<LOQ (<LOQ-0.12)	3.1
Crotonaldehyde	HPMMA	Adult	100	110 (98–130)	110 (77–180)	560
		Child	100	140 (120–160)	120 (97–180)	1600
Propylene oxide	2-HPMA	Adult	100	37 (30–45)	34 (22–59)	400
		Child	100	39 (32–46)	37 (25–58)	290

DF: Detection frequency, CI: Confidence interval, IQR: Interquartile range, NC: Not calculated, ng/L: nanogram per liter, ng/mL: nanogram per milliliter, LOQ: Limit of quantification. Geometric means and 95% confidence intervals were NC if detection frequencies were below 65%.

**Table 3:** Participant product use, housing characteristics, and activities associated with urinary log-transformed and specific gravity-adjusted 2-NAP levels.

		Β ^+^ (95% CI)	
Products Used (Yes/No)	Percent Used in Home	Adults	Children
Air Fresheners			
Any air fresheners	64%	1.80 (0.99, 3.28) *	2.16 (1.15, 4.06) *
Air freshener spray	48%	1.51 (0.82, 2.78)	1.95 (1.04, 3.63) *
Plug-in air fresheners	42%	3.69 (2.31, 5.92) *	3.08 (1.77, 5.36) *
Cleaning Products			
Number of cleaning products used		1.20 (1.04, 1.39) *	1.21 (1.04, 1.41) *
All-purpose spray or aerosol cleaners	34%	2.79 (1.61, 4.84) *	2.34 (1.27, 4.31) *
Bleach-containing products	53%	1.77 (1.02, 3.07) *	1.81 (1.02, 3.20) *
Carpet or upholstery cleaner	13%	1.63 (0.68, 3.91)	2.51 (1.05, 5.99) *
Floor cleaner	42%	1.29 (0.72, 2.32)	1.57 (0.86, 2.88)
Glass cleaner	19%	1.48 (0.70, 3.13)	1.32 (0.60, 2.90)
Toilet bowl or shower cleaner	48%	1.43 (0.78, 2.63)	1.34 (0.69, 2.59)
Disinfectant wipes	52%	0.91 (0.50, 1.63)	0.86 (0.47, 1.60)
Personal Care Products			
Perfume	64%	2.20 (1.22, 3.96) *	-
Scented body lotion	42%	1.98 (1.12, 3.5) *	-
Scented body wash	72%	1.78 (0.96, 3.32)	-
Deodorant spray	22%	1.87 (0.92, 3.78)	-
Stove used for cooking			
Electric stove	33%	1.01 (0.55, 1.86)	1.20 (0.63, 2.28)
Gas stove	64%	0.84 (0.45, 1.56)	0.67 (0.35, 1.28)
Attached garage	56%	1.15 (0.58, 2.30)	0.56 (0.29, 1.08)
Grilled food consumption	^	0.95 (0.53, 1.69)	0.83 (0.44, 1.56)

^ 53% of adults and 63% of children consumed grilled food. Only adults were asked about their personal care product use. Models were adjusted for race/ethnicity, household income, age, and cotinine levels. * represents a significant association (*p* < 0.05). **^+^** β represents the ratio of the geometric means in metabolite concentrations between product users and non-users.

## Data Availability

De-identified data from this study may be available on request subject to IRB approval. Data are not publicly available as it could compromise research participant privacy.

## Supplementary Materials

The following supporting information can be downloaded at: https://www.mdpi.com/article/10.3390/toxics14100915/s1, Table S1: Descriptive statistics of urinary metabolites (not adjusted for dilution) of PAHs and VOCs; Table S2: Descriptive statistics of creatinine-adjusted urinary metabolites of PAHs and VOCs in adults; Figure S1: Geometric means and 95% confidence intervals of PAH and VOC metabolites in BiomSPHERE and NHANES; Table S3: Geometric means and 95% confidence intervals of urinary PAH (ng/g creatinine) and VOC metabolites (ng/mg-creatinine) in U.S. population from NHANES; Figure S2: Geometric means and 95% confidence intervals of 2-NAP in Hispanic/Latino and White participants in BiomSPHERE and NHANES; Figure S3: Pearson correlation coefficients of specific gravity-adjusted and log-transformed VOCs in adults and children; Table S4: Variance in specific gravity-adjusted log-transformed urinary metabolites of PAHs and VOCs in daily urine samples collected over four days from 8 families; Table S5: Participant product use, housing characteristics, and activities associated with urinary log-transformed and specific gravity-adjusted PAH metabolites; Table S6: Participant product use, housing characteristics, and activities associated with urinary log-transformed and specific gravity-adjusted VOC metabolites; Table S7: Percent of product use in Hispanic/Latino and Not-Hispanic/Latino participants and associations between participant product use and urinary log-transformed and specific-gravity adjusted 2-NAP levels among Hispanic/Latino participants.

## Abbreviations

The following abbreviations are used in this manuscript:

PAH	Polycyclic aromatic hydrocarbon
VOC	Volatile organic compound
NAP	Hydroxynaphthalene
NHANES	National Health and Nutrition Examination Survey
SJV	San Joaquin Valley
AB 617	Assembly Bill 617
EPA	Environmental Protection Agency
BiomSPHERE	Biomonitoring component of the San Joaquin Valley Pollution and Health Environmental Research
CCAC	Central California Asthma Collaborative
LMR	Little Manila Rising
HPLC	High-performance liquid chromatography
UCSF	University of California, San Francisco
FLUO	Hydroxyfluorene
PHEN	Hydroxyphenanthrene
PYR	Hydroxypyrene
LC-MS/MS	Liquid chromatography–tandem mass spectrometry
GC/MC	Gas chromatography–mass spectrometry
CNEMA	2-cyanoethyl mercapturic acid
2-HPMA	2-hydroxypropyl mercapturic acid
3-HPMA	3-hydroxypropyl mercapturic acid
HPMMA	3-hydroxy-1-methyl-propyl mercapturic acid
PMA	Phenylmercapturic acid
ECAPCI	Electron capture atmospheric pressure chemical ionization
COT	Cotinine
3HC	Trans-3′-hydroxycotinine
LOQ	Limits of quantification
ICC	Intraclass correlations coefficient
DF	Detection frequency
CI	Confidence interval
IQR	Interquartile range
NC	Not calculated
GM	Geometric mean
CHAMACOS	Center for the Health Assessment of Mothers and Children of Salinas
SAPEP	Stockton Air Pollution Exposure Project
OEHHA	Office of Environmental Health Hazard Assessment

## Appendix A. Selection of Variables for Multivariate Regression Models

Participant demographics (age, race/ethnicity, and household income) and housing characteristics (attached garage) were obtained from the pre-sampling questionnaire. Participant activities and consumer product use were obtained from the post-sampling questionnaire and selected based on their relevance to PAH and VOC exposure. Participants were asked about their activities and product use the day before and the morning of each urine sample collection. We examined associations between the use of air fresheners (any air freshener, air freshener spray, and plug-in air freshener), cleaning products (number of cleaning products, all-purpose spray or aerosol cleaners, bleach or cleaners with bleach, carpet cleaners, floor cleaners, furniture polish, glass cleaners, bathroom cleaners, and wipes), personal care products (scented body wash, scented lotion, perfume, and deodorant spray), consumption of grilled food, type of stove used (gas vs. electric), and presence of an attached garage at participants’ residences. Only adults were asked about their personal care product use. Adult occupations were highly varied, limiting our ability to evaluate associations with metabolite levels and occupation. Furthermore, most of the first morning voids were collected on the weekends and therefore occupational exposure is less likely to influence levels of urinary metabolites.
