# Supplementary material for: Urinary Biomarkers of Exposure to Polycyclic Aromatic Hydrocarbons and Volatile Organic Compounds in San Joaquin Valley Families
Source: Toxics. 2026 Oct 16;14(10):915. doi: 10.3390/toxics14100915 (PMC13709542; doi:10.3390/toxics14100915)
Supplement: Supplementary file 1 [file toxics-14-00915-s001.zip › toxics-4555136-supplementary.pdf]

Urinary Biomarkers of Exposure to Polycyclic Aromatic Hydrocarbons and Volatile Organic Compounds in San Joaquin Valley Families: Supplementary Information

Table S1: Descriptive statistics of urinary metabolites (not adjusted for dilution) of PAHs (in ng/L) and VOCs (in ng/mL) in adults (n=64) and children (n=64) in BiomSPHERE (San Joaquin Valley, California, 2023).

Parent Compound	Metabolite	LOQ	Adult/ Child	DF (%)	Geometric Mean (95% CI)	Median (IQR)	Maximum
**Polycyclic Aromatic Hydrocarbons (ng/L)**							
Fluorene	1-FLUO	25	Adult	52	NC	26 (<LOQ-43)	1400
			Child	47	NC	<LOQ (<LOQ-51)	990
	2-FLUO	25	Adult	94	120 (94-160)	110 (64-210)	2100
			Child	92	110 (93-140)	130 (72-180)	1000
	3-FLUO	25	Adult	45	NC	<LOQ (<LOQ-42)	810
			Child	48	NC	<LOQ (<LOQ-42)	1000
Naphthalene	1-NAP	250	Adult	53	NC	250 (<LOQ-440)	7800
			Child	44	NC	<LOQ (<LOQ-590)	5900
	2-NAP	250	Adult	100	15000 (10000-21000)	17000 (4500-41000)	210000
			Child	100	15000 (11000-21000)	20000 (6800-32000)	160000
Phenanthrene	1-PHEN	25	Adult	91	79 (61-100)	67 (44-140)	2500
			Child	86	76 (59-96)	81 (39-140)	1400
	2-PHEN	25	Adult	69	49 (38-63)	42 (<LOQ-110)	1000
			Child	69	49 (38-62)	42 (<LOQ-82)	1300
	3-&4-PHEN	50	Adult	56	NC	58 (<LOQ-130)	1400
			Child	72	91 (73-110)	89 (35-150)	2500
Pyrene	1-PYR	25	Adult	88	74 (58-95)	67 (31-150)	910
			Child	88	89 (70-110)	96 (49-150)	2700
**Volatile Organic Compounds (ng/mL)**							
Acrolein	3-HPMA	1	Adult	100	260 (200-340)	310 (150-530)	2500
			Child	100	350 (280-450)	370 (190-740)	2000
Acrylonitrile	CNEMA	0.5	Adult	92	2.5 (1.7-3.7)	1.9 (1.2-3.4)	630
			Child	94	2 (1.6-2.5)	2.2 (1.2-3.1)	14
Benzene	PMA	0.1	Adult	31	NC	<LOQ (<LOQ-0.12)	3.2
			Child	31	NC	<LOQ (<LOQ-0.11)	4.8
Crotonaldehyde	HPMMA	1	Adult	100	110 (88-130)	110 (69-190)	560
			Child	100	150 (130-190)	160 (99-230)	1600
Propylene oxide	2-HPMA	1	Adult	100	35 (27-45)	32 (19-67)	510
			Child	100	43 (34-54)	45 (25-72)	450

LOQ: Limit of quantification, DF: Detection frequency, CI: Confidence interval, IQR: Interquartile range, NC: Not calculated, ng/L: nanogram per liter, ng/mL: nanogram per milliliter. Geometric means and 95% confidence intervals were NC if detection frequencies were below 65%.

Table S2: Descriptive statistics of creatinine-adjusted urinary metabolites of PAHs (in ng/g-creatinine) and VOCs (in ng/mg-creatinine) in adults (n=64) and children (n=64) in BiomSPHERE (San Joaquin Valley, California, 2023).

Parent Compound	Metabolite	Adult/ Child	DF (%)	Geometric Mean (95% CI)	Median (IQR)	Maximum
**Polycyclic Aromatic Hydrocarbons (ng/g-creatinine)**						
Fluorene	1-FLUO	Adult	52	NC	33 (<LOQ-60)	880
		Child	47	NC	<LOQ (<LOQ-49)	650
	2-FLUO	Adult	94	130 (110-160)	130 (77-200)	1500
		Child	92	110 (92-130)	120 (84-160)	670
	3-FLUO	Adult	45	NC	<LOQ (<LOQ-57)	730
		Child	48	NC	<LOQ (<LOQ-49)	670
Naphthalene	1-NAP	Adult	53	NC	360 (<LOQ-700)	7600
		Child	44	NC	<LOQ (<LOQ-630)	4300
	2-NAP	Adult	100	16000 (12000-22000)	15000 (7300-45000)	180000
		Child	100	14000 (11000-19000)	14000 (7000-40000)	100000
Phenanthrene	1-PHEN	Adult	91	86 (67-110)	86 (44-140)	1600
		Child	86	73 (58-91)	75 (43-140)	930
	2-PHEN	Adult	69	53 (43-66)	53 (<LOQ-99)	620
		Child	69	47 (38-58)	44 (<LOQ-77)	830
	3-&4-PHEN	Adult	56	NC	81 (<LOQ-120)	900
		Child	72	88 (71-110)	81 (<LOQ-130)	1600
Pyrene	1-PYR	Adult	88	80 (64-100)	82 (43-140)	590
		Child	88	85 (69-100)	80 (64-120)	1800
**Volatile Organic Compounds (ng/mg-creatinine)**						
Acrolein	3-HPMA	Adult	100	280 (230-340)	290 (210-400)	4000
		Child	100	340 (290-390)	330 (240-460)	1400
Acrylonitrile	CNEMA	Adult	92	2.7 (1.9-3.8)	1.8 (1.3-3.2)	330
		Child	94	1.9 (1.7-2.2)	1.9 (1.3-2.5)	11
Benzene	PMA	Adult	31	NC	<LOQ (<LOQ-0.18)	2.3
		Child	31	NC	<LOQ (<LOQ-0.15)	3.3
Crotonaldehyde	HPMMA	Adult	100	120 (99-140)	100 (75-160)	910
		Child	100	150 (130-170)	140 (110-180)	1400
Propylene oxide	2-HPMA	Adult	100	38 (31-46)	38 (22-56)	460
		Child	100	41 (34-49)	39 (26-58)	320

DF: Detection frequency, CI: Confidence interval, IQR: Interquartile range, NC: Not calculated, ng/g: nanogram per gram, ng/mg: nanogram per milligram. Geometric means and 95% confidence intervals were NC if detection frequencies were below 65%.

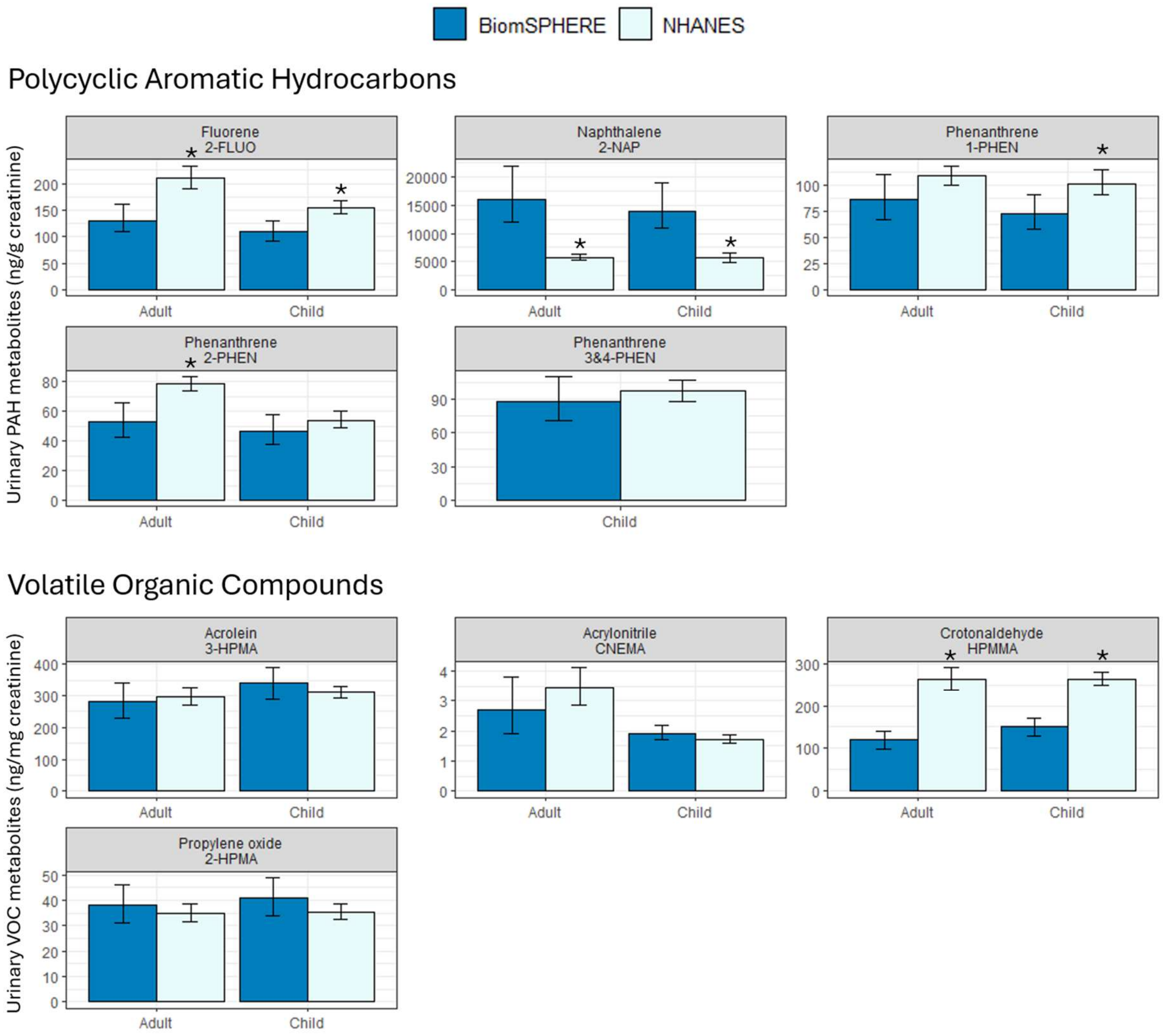

Figure S1: Geometric means (GM) and 95% confidence intervals of PAH and VOC metabolites in BiomSPHERE and NHANES. *represents a significant difference (p-value <0.05) between BiomSPHERE and NHANES. Source for 2-FLUO, 1-PHEN, 1-PYR, 3-HPMA, CNEMA, HPMMA, and 2-HPMA: NHANES 2017-2018; source for 2-NAP: NHANES 2015-2016; source for 2-PHEN and 3&4-PHEN: NHANES 2011-2012. GM and 95% CI were not calculated for 3&4-PHEN in adults and 1-PYR in adults and children because detection frequency was below 65% in BiomSPHERE and NHANES respectively.

Table S3: Geometric means and 95% confidence intervals of urinary PAH (ng/g creatinine) and VOC metabolites (ng/mg-creatinine) in U.S. population from NHANES.

				NHANES		Non-Smoking NHANES	
**Parent Compound**	**Metabolite**	**Group**	**NHANES Cycle**	**DF%**	**GM (95% CI)**	**DF%**	**GM (95% CI)**
Fluorene	2-FLUO	Adult	2017 - 2018	100%	210 (190-230)	100%	140 (130-150)
		Child		100%	150 (140-170)	100%	150 (140-170)
	3-FLUO	Adult	2017 - 2018	97%	95 (82-110)	96%	57 (51-64)
		Child		97%	73 (67-80)	97%	73 (66-80)
Naphthalene	1-NAP	Adult	2017 - 2018	99%	2000 (1700-2300)	98%	1300 (1000-1500)
		Child		98%	1200 (1000-1300)	98%	1200 (1000-1300)
	2-NAP	Adult	2015 - 2016	100%	5800 (5300-6300)	100%	4600 (4200-5000)
		Child		100%	5700 (5000-6600)	100%	5700 (4900-6600)
Phenanthrene	1-PHEN	Adult	2017 - 2018	99%	110 (100-120)	99%	96 (88-110)
		Child		99%	100 (91-110)	99%	100 (90-110)
	2-PHEN	Adult	2011 – 2012	98%	79 (74-84)	98%	66 (61-71)
		Child		95%	54 (49-60)	94%	54 (49-60)
	3&4-PHEN	Adult	2011 – 2012	97%	100 (98-110)	96%	83 (76-90)
		Child		97%	97 (88-110)	97%	97 (88-110)
Pyrene	1-PYR	Adult	2017 - 2018	60%	NC	49%	NC
		Child		64%	NC	62%	NC
Acrolein	3-HPMA	Adult	2017 - 2018	100%	300 (270-330)	100%	210 (200-230)
		Child		100%	310 (290-330)	100%	310 (290-330)
Acrylonitrile	CNEMA	Adult	2017 - 2018	81%	3.4 (2.9-4.1)	75%	1.3 (1.2-1.5)
		Child		78%	1.7 (1.6-1.9)	78%	1.7 (1.6-1.8)
Benzene	PMA	Adult	2017 - 2018	47%	NC	44%	NC
		Child		46%	NC	46%	NC
Crotonaldehyde	HPMMA	Adult	2017 - 2018	100%	260 (240-290)	100%	180 (170-200)
		Child		100%	260 (250-280)	100%	260 (250-280)
Propylene oxide	2-HPMA	Adult	2017 - 2018	96%	35 (32-39)	94%	29 (26-32)
		Child		92%	35 (33-39)	92%	35 (33-38)

DF%: Detection frequency, NC: Not calculated. Geometric means and 95% confidence intervals were not calculated if detection frequencies were below 65%. Adults are between 28 to 66 years old and children are between 3-13 years old. Non-smoking was defined as participants having serum cotinine ≤ 10 ng/mL for the 2011-2012 and urinary cotinine ≤ 91.7 ng/mL for the 2015-2016 and 2017-2018 cycles.

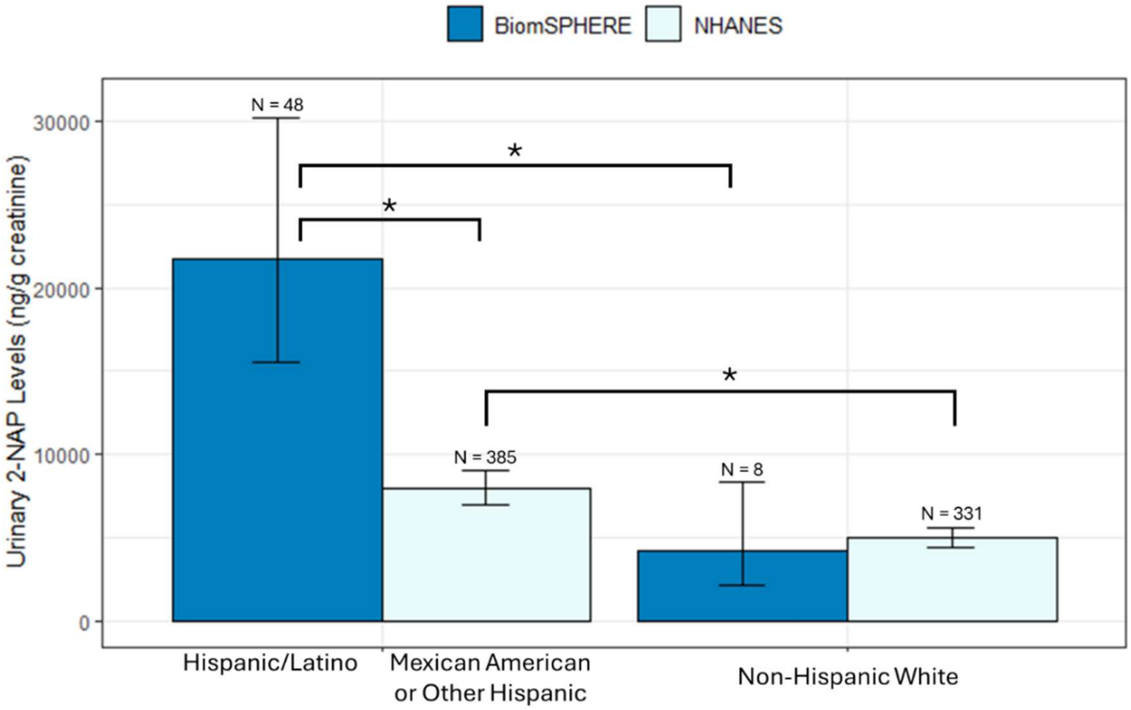

Figure S2: Geometric means and 95% confidence intervals of 2-NAP in Hispanic/Latino and White participants in BiomSPHERE and NHANES. * represents a significant association ($p < 0.05$)

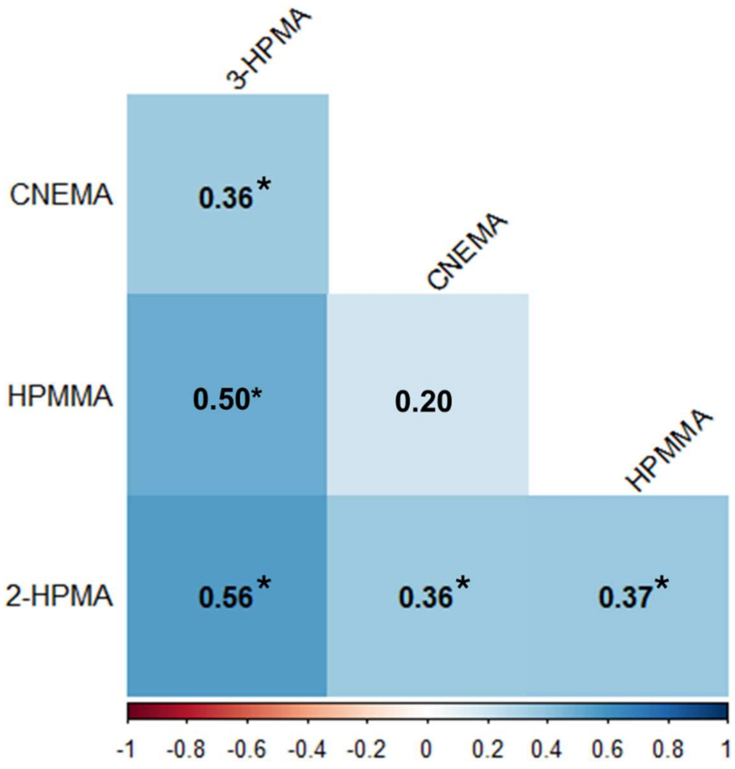

Figure S3: Pearson correlation coefficients of specific gravity-adjusted and log-transformed VOCs in adults and children. * represents a significant association (p <0.05).

Table S4: Variance in specific gravity-adjusted log-transformed urinary metabolites of PAHs and VOCs in daily urine samples collected over four days from 8 families.

	**PAHs**				**VOCs**			
	**2-FLUO**	**2-NAP**	**1-PYR**	**1-PHEN**	**HPMMA**	**2HPMA**	**3HPMA**	**CNEMA**
**Adult**								
Between	0.75	1.12	0.50	0.05	0.33	0.38	0.65	4.13
Within	0.14	0.07	0.13	0.58	0.11	0.25	0.25	0.51
ICC	**0.84**	**0.94**	**0.79**	**0.07**	**0.75**	**0.60**	**0.72**	**0.89**
**Child**								
Between	1.15e-20	0.96	0.24	0.16	0.04	0.19	0.17	0.23
Within	0.16	0.12	0.35	0.46	0.08	0.22	0.15	0.21
ICC	**7.42e-20**	**0.88**	**0.40**	**0.25**	**0.35**	**0.46**	**0.53**	**0.50**

Table S5: Participant product use, housing characteristics, and activities associated with urinary log-transformed and specific gravity-adjusted PAH metabolites.

	∑ FLUO Metabolites		∑ PHEN Metabolites		1-PYR	
	β (95% CI)		β (95% CI)		β (95% CI)	
	Adult	Child	Adult	Child	Adult	Child
**Detection Frequencies**	94%	92%	91%	86%	88%	88%
**Air Fresheners**						
Any air fresheners	0.58 (0.39, 0.88)*	1.11 (0.75, 1.63)	0.49 (0.32, 0.76)*	0.96 (0.61, 1.51)	0.62 (0.38, 0.99)*	0.95 (0.58, 1.55)
Air fresheners spray	0.68 (0.44, 1.04)	0.93 (0.64, 1.36)	0.58 (0.37, 0.91)*	0.83 (0.54, 1.28)	0.59 (0.37, 0.95)*	0.90 (0.56, 1.45)
Plug-in air fresheners	0.93 (0.61, 1.41)	0.97 (0.67, 1.4)	0.84 (0.53, 1.32)	0.86 (0.56, 1.31)	0.85 (0.53, 1.37)	0.82 (0.52, 1.31)
**Cleaning Products**						
Number of cleaning products	1.00 (0.9, 1.12)	0.92 (0.84, 1.01)	0.94 (0.84, 1.06)	0.89 (0.80, 0.99)*	0.90 (0.80, 1.02)	0.89 (0.80, 1.00)*
All-purpose spray or aerosol cleaners	0.78 (0.51, 1.2)	0.92 (0.63, 1.35)	0.68 (0.43, 1.09)	0.88 (0.56, 1.36)	0.62 (0.39, 1.00)*	0.90 (0.56, 1.45)
Bleach or cleaners with bleach	1.10 (0.73, 1.64)	0.61 (0.44, 0.83)*	0.94 (0.61, 1.47)	0.53 (0.37, 0.76)*	1.02 (0.65, 1.62)	0.66 (0.43, 1.01)*
Carpet or upholstery cleaner	0.83 (0.45, 1.55)	0.90 (0.53, 1.53)	0.79 (0.40, 1.55)	0.85 (0.46, 1.57)	0.71 (0.35, 1.43)	0.86 (0.44, 1.68)
Floor cleaner	0.97 (0.64, 1.47)	0.91 (0.63, 1.3)	0.80 (0.51, 1.25)	0.90 (0.59, 1.37)	0.63 (0.40, 1.00)*	0.67 (0.43, 1.04)
Glass cleaner	1.23 (0.73, 2.10)	0.86 (0.54, 1.36)	1.10 (0.61, 1.97)	0.74 (0.43, 1.26)	0.89 (0.49, 1.62)	0.84 (0.47, 1.51)
Toilet bowl or shower cleaner	1.30 (0.85, 2.0)	0.85 (0.58, 1.26)	1.13 (0.70, 1.82)	0.97 (0.62, 1.52)	1.01 (0.62, 1.66)	0.90 (0.55, 1.47)
Disinfectant wipes	0.99 (0.66, 1.51)	0.83 (0.58, 1.18)	0.81 (0.51, 1.27)	0.63 (0.42, 0.94)*	0.76 (0.47, 1.20)	0.63 (0.41, 0.98)*
**Personal Care Products**						
Perfume	0.76 (0.49, 1.17)	-	0.75 (0.47, 1.21)	-	0.84 (0.51, 1.38)	-
Scented body wash	1.00 (0.65, 1.52)	-	0.86 (0.54, 1.37)	-	0.81 (0.51, 1.31)	-
Scented lotion	0.94 (0.6, 1.48)	-	1.03 (0.63, 1.70)	-	0.92 (0.55, 1.53)	-
**Stove used for cooking**						
Electric stove	0.89 (0.58, 1.37)	0.67 (0.46, 0.96)*	0.89 (0.56, 1.43)	0.58 (0.38, 0.88)*	0.83 (0.51, 1.35)	0.72 (0.45, 1.15)
Gas stove	1.08 (0.70, 1.66)	1.52 (1.06, 2.19)*	1.10 (0.68, 1.77)	1.89 (1.25, 2.85)*	1.18 (0.72, 1.93)	1.58 (0.99, 2.52)*
Attached garage	0.93 (0.57, 1.51)	1.33 (0.90, 1.96)	0.83 (0.49, 1.42)	1.07 (0.68, 1.70)	0.79 (0.46, 1.38)	1.12 (0.68, 1.84)
Grilled food consumption	0.92 (0.61, 1.39)	0.83 (0.56, 1.22)	1.00 (0.64, 1.56)	0.98 (0.64, 1.51)	1.07 (0.67, 1.70)	0.89 (0.56, 1.42)

Only adults were asked about their personal care product use. Models were adjusted for race/ethnicity, household income, age, and cotinine levels. *represents a significant association ($p < 0.05$). β represents the ratio of the geometric means in metabolite concentrations between product users and non-users.

Table S6: Participant product use, housing characteristics, and activities associated with urinary log-transformed and specific gravity-adjusted VOC metabolites.

	3-HPMA		CNEMA		HPMMA		2-HPMA	
	β (95% CI)		β (95% CI)		β (95% CI)		β (95% CI)	
	Adult	Child	Adult	Child	Adult	Child	Adult	Child
Detection Frequencies	100%	100%	92%	94%	100%	100%	100%	100%
**Air Fresheners**								
Any air fresheners	0.73 (0.46, 1.18)	1.08 (0.74, 1.56)	0.57 (0.32, 1.01)*	1.18 (0.84, 1.65)	0.87 (0.62, 1.22)	0.97 (0.71, 1.34)	0.80 (0.53, 1.20)	1.22 (0.80, 1.87)
Air fresheners spray	0.81 (0.50, 1.31)	1.37 (0.96, 1.95)	0.64 (0.36, 1.16)	1.07 (0.77, 1.49)	0.90 (0.64, 1.27)	0.85 (0.62, 1.16)	0.89 (0.59, 1.34)	1.40 (0.93, 2.11)
Plug-in air fresheners	1.21 (0.76, 1.91)	1.04 (0.73, 1.49)	1.05 (0.60, 1.85)	1.22 (0.89, 1.68)	1.09 (0.78, 1.51)	1.06 (0.78, 1.43)	1.15 (0.78, 1.71)	1.12 (0.74, 1.68)
**Cleaning Products**								
Number of cleaning products	0.89 (0.79, 1.00)*	0.97 (0.88, 1.06)	0.99 (0.85, 1.14)	0.95 (0.87, 1.03)	0.96 (0.88, 1.04)	0.99 (0.91, 1.07)	0.92 (0.84, 1.02)	0.91 (0.82, 1.01)
All-purpose spray or aerosol cleaners	0.76 (0.47, 1.23)	1.10 (0.76, 1.58)	0.73 (0.40, 1.31)	0.88 (0.63, 1.23)	1.14 (0.81, 1.61)	0.88 (0.65, 1.21)	0.93 (0.62, 1.41)	1.15 (0.76, 1.75)
Bleach or cleaners with bleach	1.16 (0.74, 1.81)	1.13 (0.81, 1.57)	0.94 (0.54, 1.62)	0.86 (0.64, 1.16)	1.02 (0.74, 1.40)	0.97 (0.73, 1.29)	0.93 (0.64, 1.36)	0.83 (0.57, 1.22)
Carpet or upholstery cleaner	0.74 (0.37, 1.48)	1.03 (0.62, 1.72)	0.77 (0.33, 1.79)	0.85 (0.54, 1.35)	0.84 (0.51, 1.37)	1.35 (0.88, 2.07)	0.91 (0.51, 1.64)	0.86 (0.48, 1.53)
Floor cleaner	0.57 (0.37, 0.88)*	0.75 (0.54, 1.06)	0.82 (0.47, 1.45)	0.89 (0.65, 1.22)	0.76 (0.55, 1.05)	0.92 (0.68, 1.23)	0.79 (0.54, 1.16)	0.75 (0.51, 1.10)
Glass cleaner	0.71 (0.40, 1.27)	1.00 (0.64, 1.57)	2.25 (1.13, 4.50)*	1.22 (0.82, 1.83)	0.68 (0.45, 1.01)	0.82 (0.56, 1.19)	0.80 (0.49, 1.33)	0.60 (0.36,0.98)*
Toilet bowl or shower cleaner	0.77 (0.48, 1.23)	0.94 (0.65, 1.37)	1.01 (0.56, 1.82)	0.92 (0.65, 1.29)	0.80 (0.57, 1.13)	1.11 (0.81, 1.53)	0.77 (0.51, 1.15)	0.87 (0.57,1.34)
Disinfectant wipes	0.71 (0.45, 1.11)	0.82 (0.58, 1.16)	1.13 (0.64, 1.99)	0.84 (0.62, 1.16)	0.83 (0.60, 1.15)	0.89 (0.66, 1.19)	0.75 (0.51, 1.11)	0.65 (0.45,0.96)*
**Personal Care Products**								
Perfume	0.67 (0.42, 1.08)	-	0.59 (0.33, 1.05)	-	0.92 (0.65, 1.30)	-	0.80 (0.53, 1.20)	-
Scented body wash	1.05 (0.66, 1.68)	-	0.78 (0.44, 1.39)	-	1.28 (0.93, 1.78)	-	1.05 (0.70, 1.56)	-
Scented lotion	1.18 (0.72, 1.95)	-	0.71 (0.38, 1.3)	-	1.14 (0.80, 1.63)	-	0.91 (0.59, 1.40)	-
**Stove used for cooking**								
Electric stove	0.81 (0.50, 1.3)	0.84 (0.58, 1.20)	1.27 (0.71, 2.28)	0.82 (0.59, 1.14)	1.12 (0.79, 1.57)	1.13 (0.83, 1.54)	1.07 (0.71, 1.60)	0.81 (0.54,1.23)
Gas stove	1.22 (0.76, 1.98)	1.27 (0.88, 1.82)	0.79 (0.44, 1.42)	1.15 (0.83, 1.61)	0.80 (0.57, 1.12)	0.85 (0.62, 1.17)	0.88 (0.59, 1.33)	1.11 (0.73,1.69)
Attached garage	1.10 (0.64, 1.90)	0.79 (0.54, 1.15)	1.32 (0.68, 2.57)	0.85 (0.60, 1.19)	1.10 (0.75, 1.62)	0.78 (0.57, 1.08)	1.29 (0.82, 2.03)	1.05 (0.68,1.63)
Grilled food consumption	1.26 (0.81, 1.97)	1.04 (0.71, 1.51)	1.55 (0.90, 2.66)	0.85 (0.60, 1.19)	1.04 (0.76, 1.44)	0.87 (0.63, 1.20)	0.96 (0.65, 1.41)	1.12 (0.74,1.71)

Only adults were asked about their personal care product use. Models were adjusted for race/ethnicity, household income, age, and cotinine levels. *represents a significant association ($p < 0.05$). β represents the ratio of the geometric means in metabolite concentrations between product users and non-users.

Table S7: Percent of product use in Hispanic/Latino and Not-Hispanic/Latino participants and associations between participant product use and urinary log-transformed and specific-gravity adjusted 2-NAP levels among Hispanic/Latino participants

	**Percent Used in Home**		**β (95% CI)**	
**Products used**	**Hispanic/ Latino (N = 48)**	**Not-Hispanic/ Latino (N = 16)**	**Adults**	**Children**
**Air Fresheners**				
Any air fresheners	70%	44%	1.69 (0.80, 3.57)	1.85 (0.87, 3.96)
Air fresheners spray	56%	25%	1.23 (0.61, 2.49)	1.71 (0.84, 3.50)
Plug-in air fresheners	45%	31%	4.26 (2.57, 7.07)*	2.95 (1.57,5.56)*
**Cleaning Products**				
Number of cleaning products	-	-	1.19 (1.01, 1.41)*	1.19 (1.00, 1.41)*
All-purpose spray	33%	38%	2.25 (1.10, 4.59)*	1.77 (0.83, 3.74)
Bleach or cleaners with bleach	56%	44%	1.99 (1.03, 3.85)*	2.02 (1.03, 3.97)*
Carpet or upholstery cleaner	13%	13%	1.34 (0.46, 3.90)	1.92 (0.66, 5.52)
**Personal Care Products**				
Perfume	70%	44%	2.25 (1.09, 4.67)*	-
Scented Lotion	47%	25%	1.7 (0.86, 3.38)	-

Only adults were asked about their personal care product use. Models were adjusted household income, age, and cotinine levels. *Represents a significant association ($p < 0.05$). β represents the ratio of the geometric means in metabolite concentrations between product users and non-users.
